# Predictability of maxillary canine retraction comparing power arm and non-power arm using 24 sets of In-house clear aligner in premolar extraction case: a randomized controlled trial

**DOI:** 10.1186/s12903-025-05891-w

**Published:** 2025-05-14

**Authors:** Sawitt Eurutairat, Natnicha Vongtiang, Sakda Wonghinkong, Somchai Manopatanakul, Peerapong Santiwong, Nita Viwattanatipa

**Affiliations:** 1 Candidate Master’s Degree in Orthodontics, Faculty of Dentistry, Mahidol University, Bangkok, Thailand; 2 Private practice, Bangkok, Thailand; 3 Candidate Residency Training in Orthodontics, Faculty of Dentistry, Mahidol University, Bangkok, Thailand; 4 Department of Orthodontics, Faculty of Dentistry, Mahidol University, Bangkok, Thailand

**Keywords:** In-house clear aligner, Accuracy, Premolar extraction, Power arm, Randomized controlled trial

## Abstract

**Background:**

The bowing effect observed during premolar extractions presents a challenge in clear aligner therapy. This study aims to investigate the accuracy of maxillary tooth movement in first premolar extraction cases using the in-house clear aligner (IHCA), comparing the palatal power arm (PA) and non-power arm (control / C).

**Methods:**

Eighteen adults requiring maxillary first premolars extraction using IHCA were recruited. Using a randomized controlled trial with a split-mouth design, each patient received treatment for both PA and C. Data at the 24th IHCA comprising virtual-power arm (VPa), virtual-control (VC), actual-power arm (APa) and actual-control (AC) were measured by superimposition with pretreatment digital model, using 3D GOM Inspect software. Six types of tooth movement were assessed. Paired t-test or Wilcoxon signed-rank test was used to compare the differences between groups. Root mean square error (RMSE) as predictability was computed.

**Results:**

For the maxillary canine, there was no significant difference between the PA and C groups for all types of tooth movement except rotation. Specifically, the PA exhibited a significantly less difference in distal-in rotation compared to the control group (APa-VPa -3.54°/AC-VC -11.57°). Similarly, the RMSE of PA demonstrated better accuracy in rotation than the control (PA 7.85°/control 15.98°). In terms of anchorage, the RMSE of PA indicated greater deviation than the control in the second premolar mesial-in rotation and crown-tipping. Regarding the first molar, the RMSE of PA was mostly similar to that of the control.

**Conclusion:**

IHCA can effectively retract maxillary canines in cases involving premolar extraction. However, although palatal power arms improve the accuracy of canine rotation, no notable benefits are seen for other types of tooth movement or for anchorage control.

**Trial registration:**

Current Controlled Trials ISRCTN14020146 of the International Standard Randomized Controlled Trial. The date of registration was 16/11/2022. The trial was retrospectively registered.

## Background

Orthodontic treatment utilizing clear aligners has gained significant attention within the orthodontic profession, attributed to its aesthetic appeal, enhancement of patient comfort, and facilitation of oral hygiene maintenance. Furthermore, contemporary evidence suggests that clear aligners are effective in managing more complex malocclusions [1–3].

Initially, the overall accuracy of the aligner has been reported to be 41% in non-extraction cases (Kravitz, Kusnoto et al., 2009) [4]. Since that time, various concepts, methodologies, and adjuncts have been devised to enhance the efficacy and effectiveness of clear aligners. Nevertheless, the predictability of tooth movement, comparing actual versus predicted outcomes with aligners, remains at approximately 50% [1–3, 5].

The effectiveness of managing orthodontic cases involving premolar extractions remains one of the most challenging conditions. Several critical limitations have been encountered; for instance, incisor retraction was insufficient to reduce overjet, with alleged weaknesses in torque control [6, 7]. Specifically, reports have shown that using clear aligner therapy in extraction cases can cause a bowing effect or roller-coaster effect [8–10]. This effect is similar to the situation when a low-strength wire, such as NiTi archwire, is used for anterior teeth retraction [6, 10–12]. Additionally, using clear aligner therapy in extraction cases can lead to lingual tipping and extrusion of incisors, distal tipping and extrusion of canines, and mesial tipping and intrusion of posterior teeth, which result in anterior interference and mid-arch open bite [8, 9].

In addition, several finite studies were conducted to evaluate the predictability of tooth movement. Typically, clear aligner treatment in extraction cases caused lingual tipping and extrusion of incisors, distal tipping and extrusion of canines, and mesial tipping and intrusion of posterior teeth. [10, 11]. Thus, overcorrection has been introduced to diminish these unwanted tooth occurrences. For instance, Jiang et al. [13] reported the amount of incisor intrusion along with its retraction in order to achieve the bodily movement in vivo study. Liu et al. [10] depicted that a clear aligner would produce varying biomechanical effects across different tooth movement setups and anchorage preparation.

In terms of clinical predictability, Baldwin et al. [9], Dai et al. [14, 15], Ren et al. [16], and Feng et al. [17] assessed premolar extraction cases using the Invisalign system. They reported consistent evidence suggesting that the anticipated tooth movement was not entirely achieved following treatment. The tooth movement distance achieved was generally less than predicted, with greater crown tipping and anchorage loss [9, 14–17].

Several strategies have been suggested to counter unwanted orthodontic movement using clear aligners, such as compensatory setups, exaggerated reversed curves of Spee, movement staging, attachments, inter-arch elastics, etc. [2, 18–20]. For instance, Womack [21] and Gaffuri et al. [22] recommended using power arms attached to the canines alongside 3⁄16" elastics on the first-molar buttons to control canine root angulation in a first premolar extraction case using Invisalign.

As digital dentistry evolves, In-house laboratory procedures and In-house clear aligners (IHCA) have gained popularity among orthodontists [8, 23–25]. However, substantial studies evaluating the effectiveness of IHCA, particularly their accuracy in extraction cases, are still lacking [26]. Only one paper by Jaber et al. [27] reported the use of IHCA in cases involving the extraction of four first premolars, comparing treatment outcomes using the PAR index between clear aligners and fixed appliances at extraction sites. Therefore, our research question aims to evaluate the accuracy of tooth movement in premolar extraction cases using IHCA.

## Aim and hypothesis

This randomized controlled clinical trial (RCT) aimed to determine the predictability of IHCA by comparing virtual and actual tooth movement using 24 pieces of IHCA in adults who required maxillary premolar extraction, focusing on the maxillary canine and anchorage. Additionally, we compared the predictability of tooth movements between the side using a palatal power arm (PA) and the non-power arm (control/C) groups.

## Methods

### Trial design and setting

This study was a single-center randomized controlled trial with a split-mouth allocation ratio of 1:1. It was approved by the Institutional Review Board (IRB), Faculty of Dentistry and Faculty of Pharmacy (DTPY-IRB), Mahidol University, with Clinical Registry reference number ISRCTN 14020146 (retrospective) of the International Standard Randomized Controlled Trial.

### Sample size calculation

The Statulator website [28] was used to calculate the sample size for the paired category. To achieve a power of 80% and a significance level of 5% (two-sided) for detecting a mean difference of 0.37 mm between pairs, with a standard deviation of 0.50 mm [16], a minimum sample size of 18 patients was required for this study. Ultimately, 21 volunteers were enlisted to ensure adequate coverage.

### Randomization

**Fig. 1:**
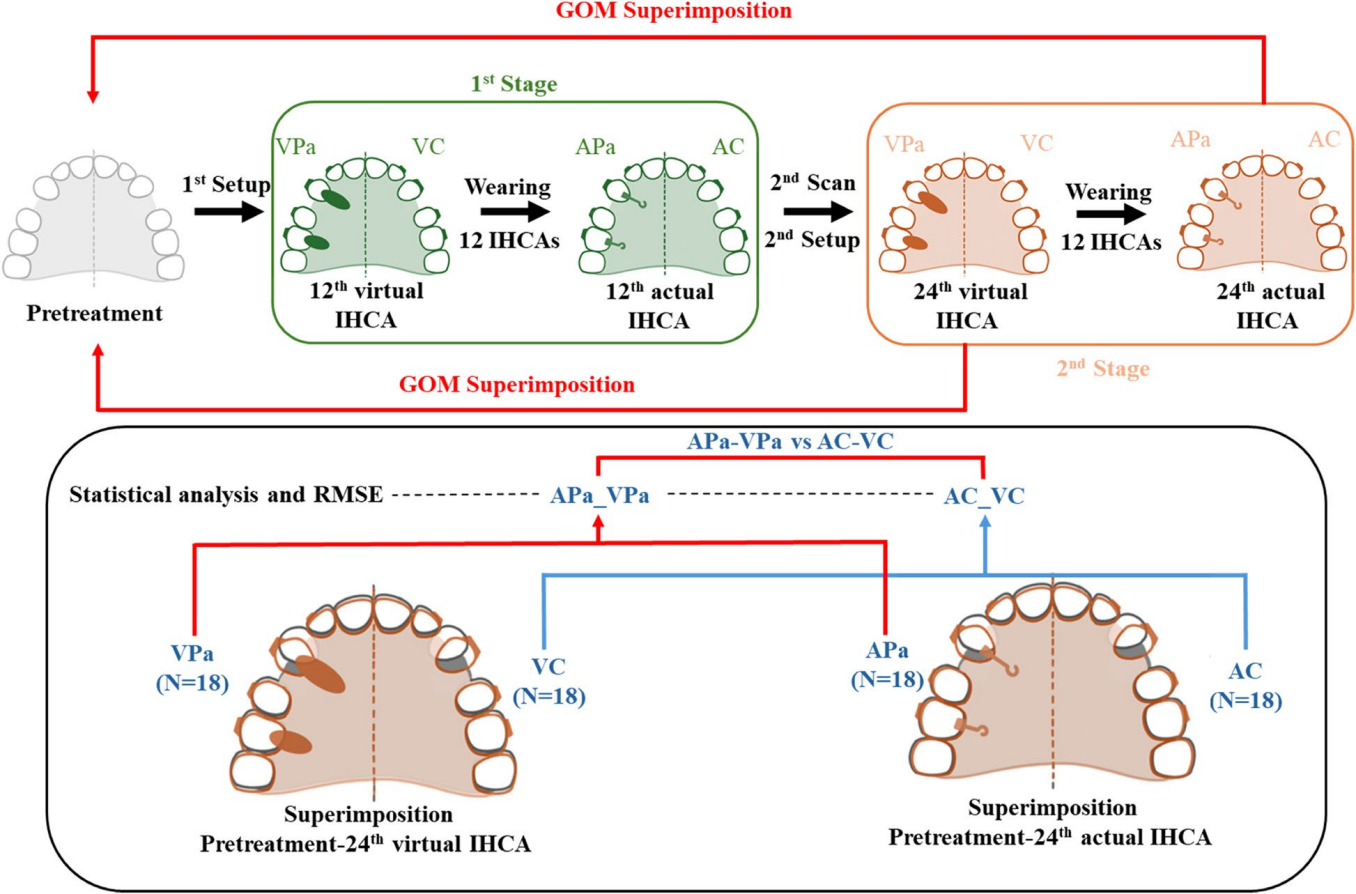
Research design

An online random generator was used to randomize the allocation of the PA side. This process took place before the experiment began, carried out by residents who had no clinical involvement in the study. Given the visibility of PA, it was not feasible to blind the patients and research operators. The research design for this study is illustrated in Fig. 1.

### Participants and eligibility criteria

IHCA patients were enrolled in this clinical trial from 2019 to 2022 at the Orthodontic Clinic, Faculty of Dentistry, Mahidol University. The inclusion and exclusion criteria are detailed in Table 1.

**Table 1:** Inclusion and exclusion criteria

**Inclusion**	**Exclusion**
1) Aged 18 years and above.	1) Moderate to severe rotation of maxillary canine position.
2) Angle Class I or II division 1 with proclination and/or protrusion of maxillary incisors.	2) Asymmetrical position of maxillary right and left canine.
3) Upper arch showed no to mild dental crowding.	3) Absence of maxillary permanent teeth except 3^rd^ molars.
4) Extraction of the maxillary first premolars.	4) Poor cooperation or compliance.
	5) Patients refuse treatment, for instance, going aboard.
	6) Pathologies. i. Presence of systemic illnesses and/or bone related diseases. ii. On medication such as taking Bisphosphonate drugs. iii. Pregnancy. iv. Hypercementosis and/or any dental anomalies. v. Periodontal diseases

### Pretreatment orthodontic protocol

Initial orthodontic records for pretreatment were collected. An iTero scanner (Align Technology, Inc., San Jose, CA) was utilized to generate the intraoral scan files. Specifically, all patients were scanned prior to treatment and before stage 2 (Table 2).

**Table 2:** Compensation tooth set-up protocol of each tooth in maxillary arch for 1 stage of clear aligner setup

Tooth	Linear movement	Angulation	Extrusion/Intrusion
**Stage 1** (1st – 12th aligners)			
Canine	Distalized 3 mm	Distal root tip 8°	0 mm
2nd premolar	-	Mesial root tip 7°	Ext 0.7 mm
1st molar	-	Mesial root tip 5°	Ext 0.5 mm
**Stage 2** (13th – 24th aligners)			
Canine	Distalized 3 mm	Distal root tip 8°	0 mm
2nd premolar	-	Mesial root tip 7°	Ext 0.7 mm
1st molar	-	Mesial root tip 5°	Ext 0.5 mm

Abbreviation: *Ext* Extrusion

### Laboratory workflow

IHCA fabrication was divided into 2 stages. The first stage was1^st^-12th IHCA, and the latter stage was 13th-24th IHCA (Table 2). For model preparation, the STL-scanned file was imported into Ortho-Analyzer software (3Shape, Copenhagen, Denmark). Then, the teeth were segmented in preparation for model setup.

### Virtual tooth movement

For each stage, the distalization of the maxillary canines was set sequentially to close the extraction spaces by moving them approximately 3 mm/stage.

In both stages, compensation protocols were implemented (Table 2) to reduce unwanted tooth movements, achieve maximum anchorage, and reduce the bowing effect [6, 12].

The virtual tooth movements were adjusted from the protocol proposed by Lombardo et al. [29]. The laboratory protocols are shown in Table 3.

**Table 3:** Laboratory protocol of In-house clear aligner treatment

Laboratory Protocol	
Linear movement in combined A-P/ Vertical/ Transverse direction	0.50 mm / model
Mesiodistal tip	Not exceeding 1° / aligner
$\text{Rotation}$	Not exceeding 1 $^\circ$/ aligner
Torque	Not exceeding 0.5° / aligner
Printing Protocols	Each model was used to construct 2 aligners with different in thickness - 0.5 mm and 0.75 mm Stage 1: 6 printed models and 1 model template for attachment placement Stage 2: 6 printed models and 1 model template for attachment placement
**Clinical Protocol**	
Auxiliaries (Stage1)	- Palatal power arm 12 mm in length on experiment canine 8 mm in length on experiment first molar - Super-elastic power chains on experimental side (80-100 g of force) - Buttons UL3/ UR3/ LL6/ LR6 - Intermaxillary elastics 3/16″ 2 oz
Auxiliaries (Stage2)	- Palatal power arm 12 mm in length on experiment canine 8 mm in length on experiment first molar - Super-elastic power chains on experimental side (80-100 g of force) - Buttons UL3/ UR3/ LL5/ LR5/ LL6/ LR6 - Intermaxillary elastics 3/16″ 2–3.5 oz
Instruction (Both stages)	-Wearing at least 22 h a day -Aligners change every 1 week/ piece -Using chewy twice daily -Follow up every 6–8 weeks

**Fig. 2:**
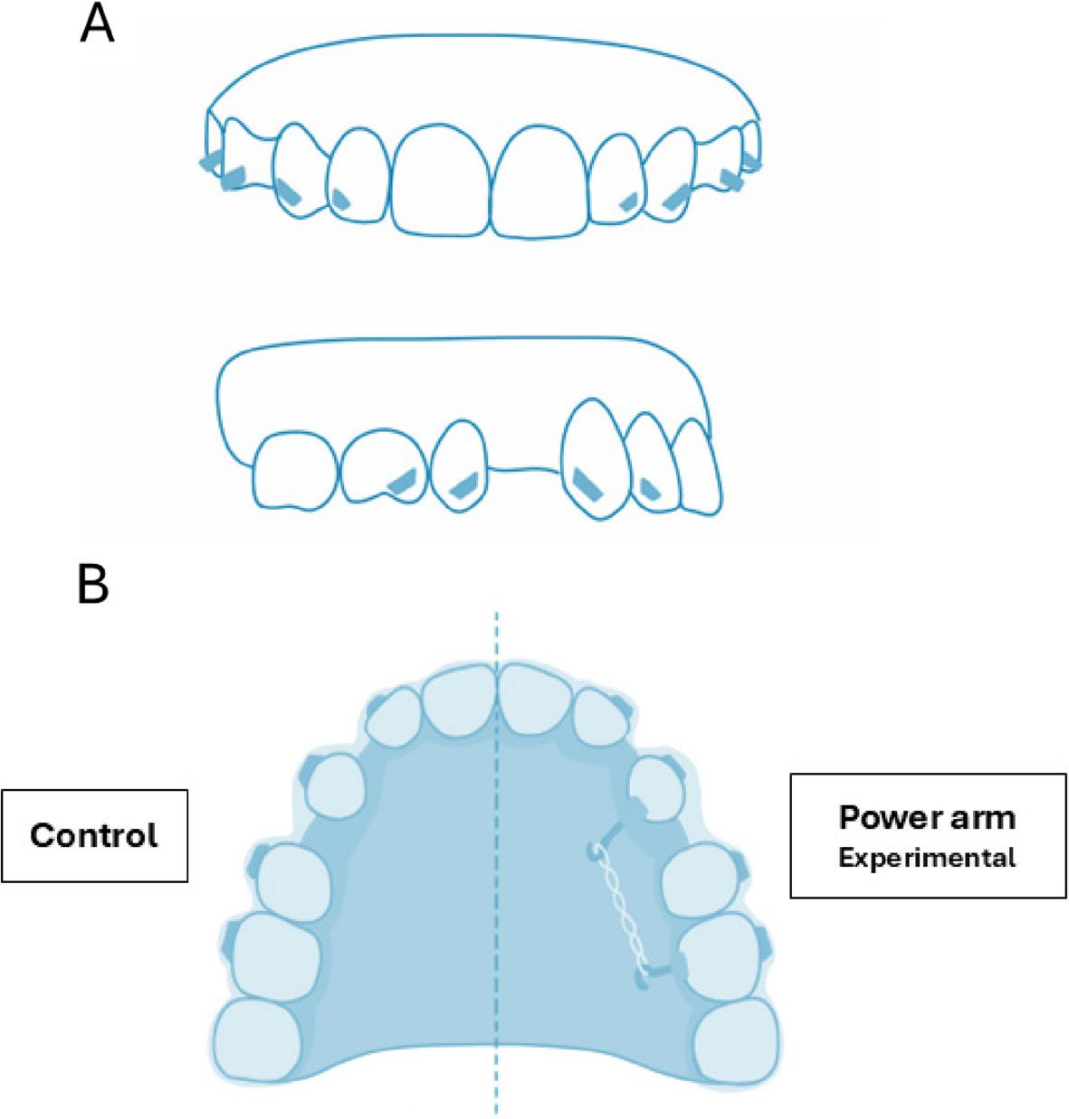
A: Location of triangular prism-shaped attachments. B: In-house clear aligner (IHCA) and the power arm (PA)

Triangular prism-shaped attachments were placed on the maxillary lateral incisors, canines, second premolars, and first molars (Fig. 2). Button cut-outs were designed on the maxillary canines and mandibular first molars.

For model printing, the virtual setup of each stage was divided into six models and one template model for the attachments and PA bonding. Digital models were printed in a 20° oblique orientation using a Formlabs 3D printer and a photopolymer resin designed for dental models (Formlabs V4, Somerville, USA).

Each printed 3D model was thermoformed with 0.5 and 0.75 mm thermoplastic sheets [30](PET-G:3A MEDES, Korea) using a pressure molding device (Biostar®; Scheu Dental, Germany).

### Clinical procedure

#### Attachment and power arm bonding (Fig. 2A, 2B)

Metal PA was placed on the palatal cervical 1/3 area of the canine and the ipsilateral 1st molar on the experimental side. The attachments were bonded. Consequently, buttons were bonded to the maxillary canines and mandibular first molars to promote the extrusion of lower molars and strengthen anchorage. Both maxillary first premolars were extracted on the same day. IHCA was delivered within one week of extraction. A super elastic power chain (TOMY Inc., Japan) was placed between the PA of the maxillary canine and molar. Details of our clinical protocols are described in Table 3.

#### Deviation analysis

**Fig. 3:**
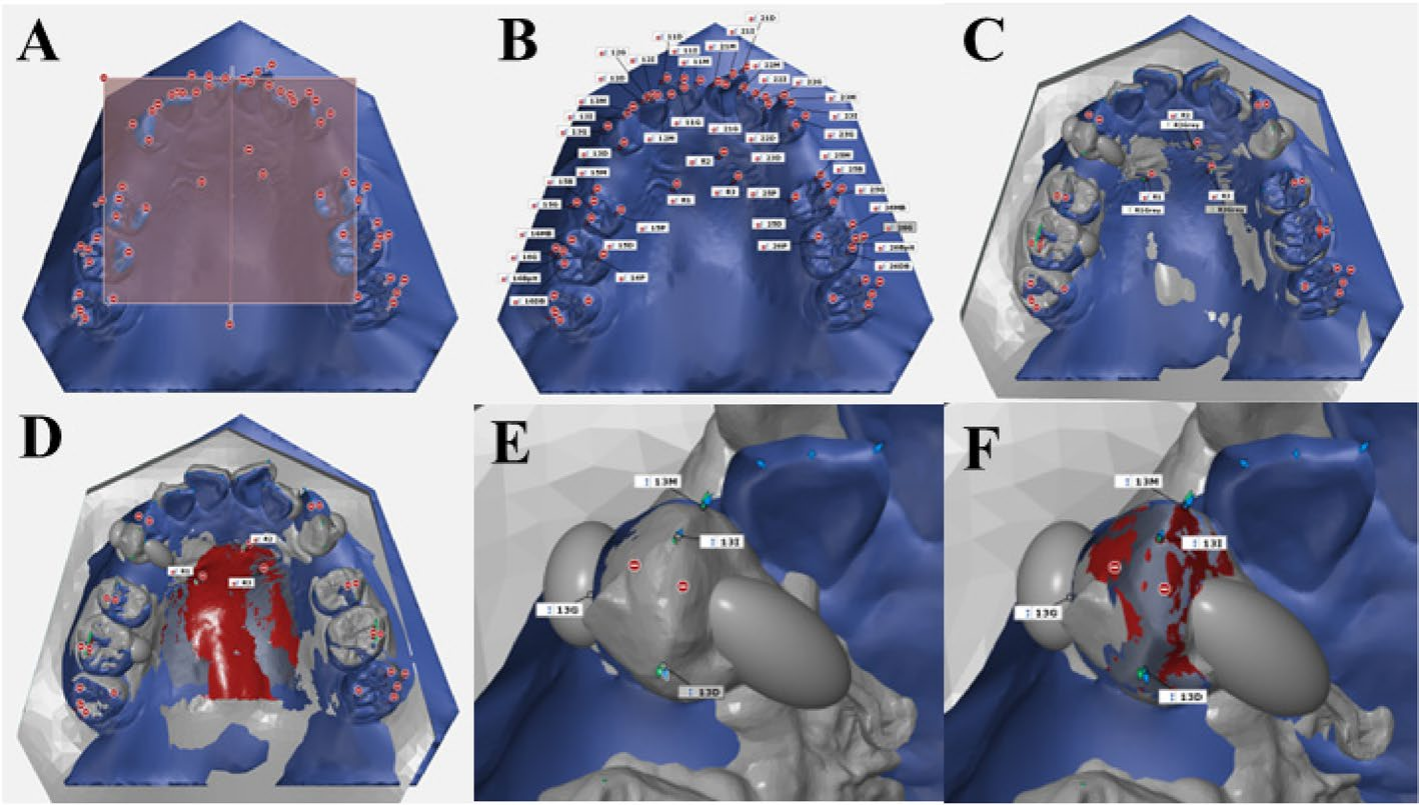
Superimposition method using GOM inspect suite. The master files were imported to GOM inspect suite software.**A**. A local coordinate system was constructed by fitting dental planes on the master model. **B**. Each point was selected and defined. **C**. Superimposition of the actual model with the master file, initially using the 3-points alignment method. **D**. Followed by the local best-fit function at stable palatal area. **E**. Superimposition of the canine firstly, by using the Geometric element method. **F**. Then superimposition of the canine by local best-fit. Thereafter, the software would automatically transfer of the points from master file and link to the actual file

STL file data of both virtual and actual were collected at pretreatment and at the 24th IHCA. Deviation analysis was conducted to assess the accuracy of the clear aligners employing the GOM Inspect Suite software (Carl Zeiss GOM Metrology, Germany). Superimposition between virtual and actual digital models, and measurement protocols were adapted from prior studies and are revealed in the following steps (Fig. 3) [31, 32]. The primary and secondary outcomes, along with their respective definitions and abbreviations, are outlined in Table 4.

**Table 4:** Definition and abbreviation terms of parameters and outcome measurement in maxillary model superimposition

Parameters	Definition and Abbreviation	
**Tooth**		- Canine - Second premolar - First molar
**Axis** **referral**		X = Transverse Y = Occluso-gingival Z = Antero-posterior
**Points** **on model**	Palate	Palate references area of 3 points (R1, R2, and R3)
	Canine	M = mesial point angle I = cusp tip D = distal point angle G = center of gingival surface at buccal
	Second Premolar	B = buccal cusp MB = mesio-buccal point angle DB = disto-buccal point angle G = center of gingival surface at buccal
	First Molar	B = occlusal point of buccal groove MB = mesio-buccal cusp tip DB = disto-buccal cusp tip G = center of gingival surface at buccal
**Angulation on model**	Tip	Angle between two B/I – G lines in YZ Axis
	Torque	Angle between two B/I – G lines in XY Axis
	Rotation	Angle between two M—D lines in XZ Axis
**Primary** **Outcomes**	Canine, Second Premolar, First Molar	RMSE = Root means square error Mesio-Distal Displacement Extrusive-Intrusive Displacement Bucco-Lingual Displacement Tipping Torque Rotation

#### Model superimposition

The virtual pretreatment and the 24th IHCA digital model for each patient were superimposed in STL format by a single operator using GOM software. Details regarding points and angulations on the models are presented in Table 4. The superimposition methods are outlined in the legend of Fig. 3. Measurements were taken for the maxillary canine, second premolar, first molar, and as follows:

#### Linear measurements (Table 4, Fig. 4A)

**Fig. 4:**
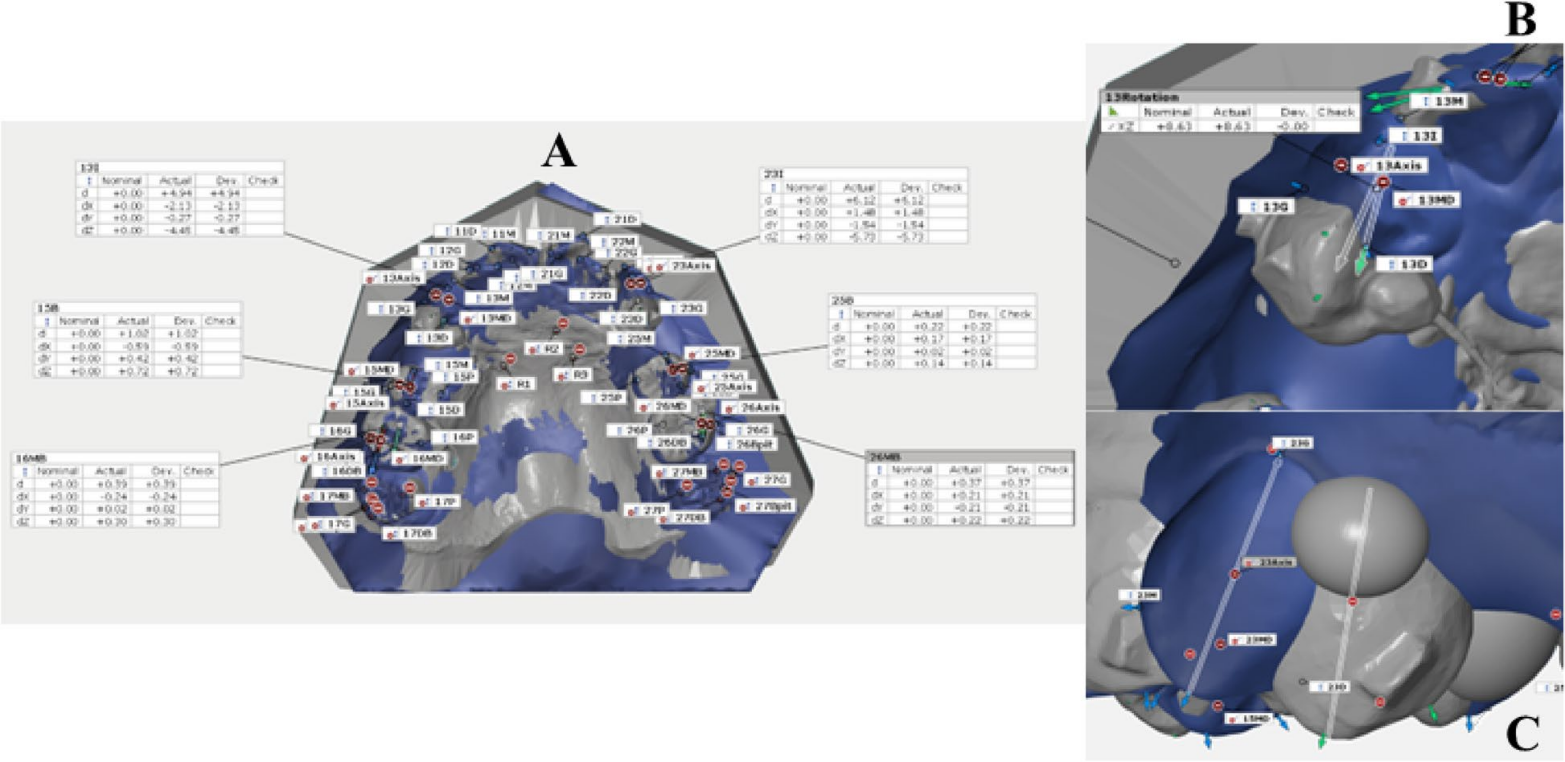
Measurement of point deviation (dX, dY, dZ) and tooth angulation between virtual and actual models were measured. A, Linear measurements; B, angle between mesio-distal lines; C, tip and torque angles between the axis lines

An outcome assessor performed measurement readouts. The virtual and actual distance changes in tooth position in the mesiodistal, intrusive-extrusive, and buccolingual directions on both sides were recorded.

#### Angular measurements (Table 4, Fig. 4B and C)

Angular measurements included the mesio-distal and bucco-lingual angulations of the tooth axis relative to the reference plane, as well as the tip, rotation, and torque angles. All measurements were recorded in an Excel spreadsheet for statistical analysis.

#### Root mean square error (RMSE)

RMSE represents accuracy in our study. The equation was as follows:

$$\mathrm{RMSE}=\mathrm{sqrt}\;\left[\left(\sum\left(\mathrm{Ai}-\mathrm{Vi}\right)^2\right)/\mathrm n\right]$$

The sum of the squared differences between the actual PA and virtual PA values (APa–VPa) and between the actual control and virtual control values (AC–VC) was divided by the number of observations, with the square root of the result yielding the RMSE. For indirect comparisons of RMSE between PA and control, a distance value greater than 0.50 mm and an angle greater than 1.5 $^\circ$ would be interpreted as a difference.

### Statistical analysis

Statistical analyses were conducted using SPSS software (version 22.0; IBM, NY, USA). The Shapiro–Wilk normality test and histograms were employed to assess the normality of the distributions. Paired t-tests or Wilcoxon signed-rank tests were utilized to examine significant differences in tooth movement changes between the two groups.

### Error analysis

Dahlberg's error formula was employed to conduct error analysis on six randomly selected pairs of digital models.

## Results

**Fig. 5:**
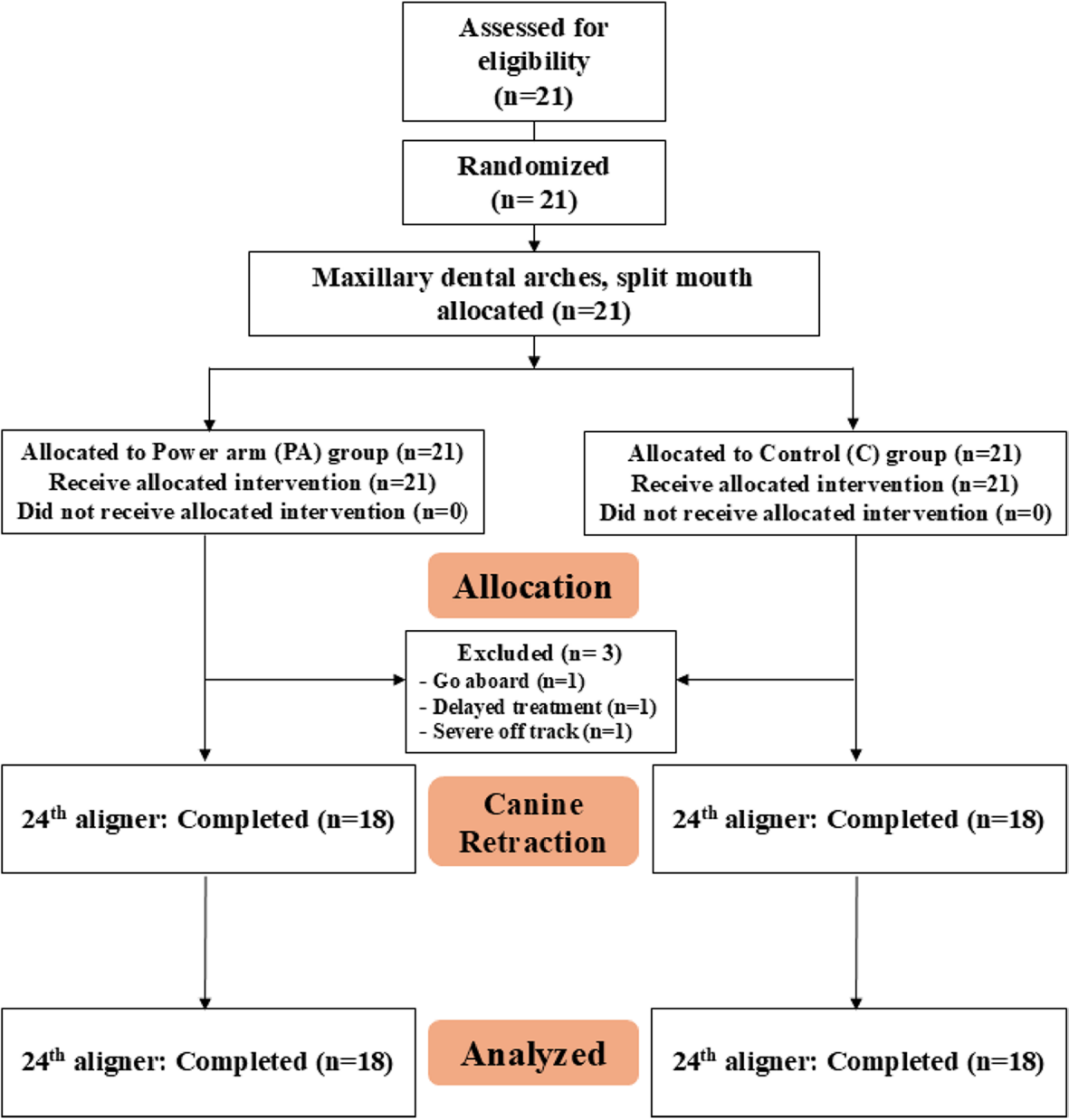
CONSORT flow chart of patient participation

The CONSORT flowchart is shown in Fig. 5. Initially, 21 patients were enrolled; however, 3 patients dropped out. The final number of participants analyzed was 18 out of 21 (85.7%).

### Patient characteristics

The demographic data of all patients is presented in Table 5. Six patients were classified as Angle’s class I, while 12 patients were classified as Angle’s class II. Overall, the mean maxillary crowding was -3.42 ± 1.50 mm, the overjet was 6.10 ± 1.69 mm, and the overbite was 3.70 ± 1.86 mm.

**Table 5:** Patient Characteristics

Parameter	Mean ± SD / Frequency
Gender	Female:Male – 17:1
Age	23.46 ± 4.62 years old
Skeletal type I Skeletal type II	3 cases 15 cases
Angle’s Classification	
- Class I - Class II div 1	6 cases 12 cases
Crowding maxillary arch	-3.42 ± 1.50 mm
Overjet Overbite	6.10 ± 1.69 mm 3.70 ± 1.86 mm
Extraction pattern	
Upper 4 s: Upper 4 s-Lowers 4 s/5 s: Upper 4 s-:	4 cases 12 cases 2 cases
Lower fixed appliance	6 cases
Total number of aligners	12 Models (24 aligners)
Treatment time	276.8 ± 58.4 days or 38.8 ± 7.9 weeks
Off-track amount	
Stage1	
None:	1 case
Mild (≤0.5 mm)	8 cases
Moderate (0.5-1 mm)	6 cases
Severe (> 1 mm)	3 cases
Stage2	
None:	7 cases
Mild (≤ 0.5 mm)	7 cases
Moderate (0.5-1 mm)	3 cases
Severe ( > 1 mm)	1 case
Off track management:	
Stage1	
None	9 cases
Back track	
1 time	4 cases
2 time	2 cases
Elastic traction	3 cases
Stage2	
None	12 cases
Back track	0 case
Elastic traction	6 cases

### Maxillary canine distalization (Table 6)

**Table 6:** Maxillary canine movement (Pre-treatment to 24 aligners); Comparison of predicted (virtual) and achieved (actual) tooth movements in all directions. Results of paired T-test or Wilcoxon signed-rank test (*N* = 18)

*Movement*	*Virtual* *Mean* ± *SD* *(95%CI)*		*Actual* *Mean* ± *SD* *(95%CI)*		P*-value*		*Difference* *Mean* ± *SD* *(95%CI)*		*P-value* *Difference*	*RMSE*	
	***VPa***	***VC***	***APa***	***AC***	***VPa_APa***	***VC_AC***	***APa-VPa***	***AC-VC***		***PA***	***C***
Distalization(mm) = + Mesialization(mm) = -	4.51 ± 0.83 *(4.10–4.92)*	4.67 ± 0.85^#^ *(4.24–5.09)*	4.70 ± 1.10 *(4.16–5.24)*	4.99 ± 0.92 *(4.54–5.45)*	0.204	0.033^*^	0.19 ± 0.61 *(-0.11–0.49)*	0.33 ± 0.60 *(0.26–0.63)*	0.26	0.62	0.67
Extrusion (mm) = + Intrusion (mm) = -	0.70 ± 1.08 *(0.16–1.24)*	0.65 ± 0.83 *(0.24–1.06)*	0.47 ± 0.85 *(0.04–0.89)*	0.39 ± 0.75 *(0.02–0.77)*	0.124	0.04^*^	-0.23 ± 0.61^#^ *(-0.54–0.07)*	-0.25 ± 0.48 *(-0.49- -0.01)*	0.88^#^	0.64	0.53
Buccal (mm) = + Lingual (mm) = -	2.05 ± 0.86 *(1.63–2.48)*	2.09 ± 0.77 *(1.71–2.48)*	2.11 ± 0.93^#^ *(1.65–2.57)*	2.05 ± 0.80 *(1.65–2.44)*	0.601^#^	0.381	0.06 ± 0.29 *(-0.09–0.20)*	-0.05 ± 0.23 *(-0.16–0.06)*	0.35	0.29	0.22
Tip (°)											
MCT = +	-0.67 ± 5.73	1.80 ± 5.65	-11.10 ± 3.12^#^	-8.80 ± 3.97	0.000^#^	0.000^*^	-10.43 ± 5.37	10.60 ± 4.58	0.91	11.66	11.50
DCT = -	*(-3.52–2.18)*	*(-1.02–4.61)*	*(-12.65- -9.55)*	*(-10.78- -6.83)*			*(-13.10- -7.76)*	-*(-12.88- -8.32)*			
Torque (°)											
BCT = +	*-2.33 ± 4.68*	*-4.16 ± 4.66*	*-0.70 ± 4.20*	*-0.89 ± 3.10*	0.236	0.019^*^	*1.63 ± 5.63*	3.28 ± 5.37	0.386	5.71	15.98
LCT = -	*(-4.65–0.00)*	*(-6.48- -1.84)*	*(-2.78–1.39)*	*(-2.43–0.66)*			*(-1.17–4.43)*	*(0.61–5.95)*			
Rotation (°)											
M-in = +	-0.51 ± 6.03^#^	-6.43 ± 9.70	-4.05 ± 7.28	-18.00 ± 8.62	0.039^#,*^	0.000^*^	-3.54 ± 7.22	-11.57 ± 11.35^#^	0.006^#,*^	7.85	15.98
D-in = -	*(-3.51–2.49)*	*(-11.25- -1.61)*	*(-7.66- -0.43)*	*(-22.28- -13.71)*			*(-7.12–0.05)*	*(-17.21- -5.92)*			

*Abbreviation: VPa* power arm side of virtual model, *VC* control side of virtual model, *APa* power arm side of actual model, *AC* control side of actual model, *APa-VPa* Difference between actual and virtual model on power arm, *VC-AC* Difference between actual and virtual model on control

Favorable movement = + (Distalization, Extrusion, Buccal, Mesial Crown Tip (MCT), Buccal Crown Torque (BCT), Mesial-in rotation)

Non-Favorable movement =—(Mesialization, Intrusion, Lingual, Distal Crown Tip (DCT), Lingual Crown Torque (LCT), Distal-in rotation)

^#^non- parametric

^*^ Statistically significant at *p* < 0.05

The difference in values between virtual and actual distalization indicated that there were no significant differences observed between the PA group (APa-VPa of 0.19 ± 0.61 mm) and the control group (AC-VC of 0.33 ± 0.60 mm). Additionally, the RMSE showed that the PA group (0.62 mm) had comparable accuracy to the control group (0.67 mm).

### Maxillary canine tipping (Table 6)

The difference in values of distal crown tipping between virtual and actual, there was no significant differences observed between the PA (APa-VPa -10.43 ± 5.37°) and control group (AC-VC -10.60 ± 4.58°). Additionally, the RMSE indicated that the PA (11.66°) showed comparable accuracy with the control group (11.50°).

### Maxillary canine rotation (Table 6)

The difference in values of distal-in rotation of canine between virtual and actual showed significant differences between the PA (APa-VPa -3.54 ± 7.22°) and control group (AC-VC -11.57 ± 11.35°). Additionally, the RMSE indicated that the PA group (7.85°) showed half less deviation than the control group (15.98°).

### Maxillary premolar mesialization (Table 7)

**Table 7:** Maxillary premolar movement (Pre-treatment to 24 aligners); Comparison of predicted (virtual) and achieved (actual) tooth movements in all directions. Results of paired T-test or Wilcoxon signed-rank test (*N* = 18)

*Movement*	*Virtual* *Mean* ± *SD* *(95%CI)*		*Actual* *Mean* ± *SD* *(95%CI)*		P-*value*		*Difference* *Mean* ± *SD* *(95%CI)*		P*-value* *Difference*	*RMSE*	
	***VPa***	***VC***	***APa***	***AC***	***VPa_APa***	***VC_AC***	***APa-VPa***	***AC-VC***		***PA***	***C***
Distalization(mm) = + Mesialization(mm) = -	0.01 ± 0.67 *(-0.32–0.35)*	-0.08 ± 0.52 *(-0.34–0.18)*	-0.60 ± 0.49 *(-0.84- -0.35)*	-0.59 ± 0.59 *(-0.88- -0.29)*	0.000^*^	0.001^*^	-0.61 ± 0.58 *(-0.90- -0.32)*	-0.51 ± 0.54 *(-0.78- -0.24)*	0.43	0.83	0.73
Extrusion (mm) = + Intrusion (mm) = -	0.33 ± 0.56 *(0.05–0.61)*	0.41 ± 0.45 *(0.19–0.63)*	-0.29 ± 0.38^#^ *(-0.48- -0.10)*	-0.06 ± 0.40 *(-0.26–0.14)*	0.000^*^	0.000^*^	-0.62 ± 0.53^#^ *(-0.88- -0.35)*	-0.47 ± 0.30 *(-0.62- -0.32)*	0.151	0.80	0.56
Buccal (mm) = + Lingual (mm) = -	0.25 ± 0.29 *(0.11–0.40)*	0.13 ± 0.44 *(-0.09–0.35)*	0.02 ± 0.43 *(-0.20–0.23)*	-0.15 ± 0.42 *(-0.36–0.06)*	0.000^*^	0.000^*^	-0.24 ± 0.23 *(-0.36- -0.13)*	-0.28 ± 0.24 *(-0.40- -0.16)*	0.66	0.32	0.36
Tip (°)											
DCT = +	3.90 ± 3.50	3.97 ± 2.71	-1.34 ± 2.64^#^	0.15 ± 2.72	0.000^#,*^	0.000^*^	-5.24 ± 3.47	-3.82 ± 2.77	0.064	6.23	4.67
MCT = -	*(2.15–5.64)*	*(2.62–5.32)*	*(-2.66- -0.03)*	*(-1.20–1.50)*			*(-6.96- -3.51)*	*(-5.19- -2.44)*			
Torque (°)											
BCT = +	*-0.02 ± 2.64*	*-0.39 ± 2.60*	*-0.84 ± 2.30*	*-0.41 ± 2.31*	0.32	0.982	*-0.82 ± 3.38*	*-0.02 ± 3.62*	0.510	3.39	3.52
LCT = -	*(-1.33–1.30)*	*(-1.68–0.90)*	*(-1.98–0.31)*	*(-1.56–0.74)*			*(-2.50–0.86)*	*(-1.82–1.78)*			
Rotation (°)											
D-in = +	*0.80 ± 4.42*	*1.32 ± 2.10*	*0.78 ± 3.43*	*-0.29 ± 2.24*	0.983	0.001^*^	*-0.02 ± 4.73* ^*#*^	*-1.62 ± 1.74*	0.25^#^	4.60	2.34
M-in = -	*(-1.40–3.00)*	*(0.28–2.37)*	*(-0.93–2.48)*	*(-1.41–0.82)*			*(-2.38–2.33)*	*(-2.48- -0.75)*			

Abbreviation: VPa = power arm side of virtual model, VC = control side of virtual model, APa = power arm side of actual model, AC = control side of actual model, APa-VPa = Difference between actual and virtual model on power arm, VC-AC = Difference between actual and virtual model on control

Favorable movement = + (Distalization, Extrusion, Buccal, Distal Crown Tip (DCT), Buccal Crown Torque (BCT), Distal-in rotation)

Non-Favorable movement =—(Mesialization, Intrusion, Lingual, Mesial Crown Tip (MCT), Lingual Crown Torque (LCT), Mesial-in rotation)

^#^non- parametric

^*^Statistically significant at *p* < 0.05

The difference in values between virtual and actual premolar mesialization, there was no significant differences between the PA (APa-VPa -0.61 ± 0.58 mm) and control (AC-VC -0.51 ± 0.54 mm). The RMSE indicated the PA group (0.83 mm) demonstrated similar deviation with the control group (0.73 mm).

### Maxillary premolar tipping (Table 7)

The difference in values between virtual and actual mesial crown tipping showed no significant differences between the PA (APa-VPa -5.24 ± 3.47°) and control group (AC-VC -3.82 ± 2.77°). The RMSE indicated the PA group (6.23°) was less accurate than the control group (4.67°).

### Maxillary premolar rotation (Table 7)

The difference in values of mesial-in rotation of premolar between virtual and actual, showed no significant differences observed between the PA (APa-VPa -0.02 ± 4.73°) and control (AC-VC –1.62 ± 1.74°). However, the RMSE indicated the PA (4.60°) showed less accuracy than the control group (2.34°).

### Maxillary molar mesialization (Table 8)

**Table 8:** Maxillary molar movement (Pre-treatment to 24 aligners); Comparison of predicted (virtual) and achieved (actual) tooth movements in all directions. Results of paired T-test or Wilcoxon signed-rank test (*N* = 18)

*Movement*	*Virtual* *Mean* ± *SD* *(95%CI)*		*Actual* *Mean* ± *SD* *(95%CI)*		P*-value*		*Difference* *Mean* ± *SD* *(95%CI)*		P*-value* *Difference*	*RMSE*	
	***VPa***	***VC***	***APa***	***AC***	***VPa_APa***	***VC_AC***	***APa-VPa***	***AC-VC***		***PA***	***C***
Distalization(mm) = + Mesialization(mm) = -	0.13 ± 0.58 *(-0.16–0.42)*	-0.08 ± 0.37 *(-0.26–0.10)*	-0.45 ± 0.48 *(-0.69- -0.21)*	-0.60 ± 0.53 *(-0.86- -0.34)*	0.001^*^	0.002^*^	-0.58 ± 0.59 *(-0.87- -0.28)*	-0.52 ± 0.59 *(-0.81- -0.23)*	0.679	0.81	0.77
Extrusion (mm) = + Intrusion (mm) = -	0.48 ± 0.61 *(0.17–0.78)*	0.49 ± 0.35 *(0.32–0.66)*	-0.16 ± 0.52 *(-0.42–0.10)*	-0.03 ± 0.36 *(-0.21–0.15)*	0.000^*^	0.000^*^	-0.63 ± 0.49 *(-0.88- -0.39)*	-0.52 ± 0.28 *(-0.66- -0.38)*	0.278	0.79	0.58
Buccal (mm) = + Lingual (mm) = -	0.41 ± 0.46 *(0.18–0.64)*	0.40 ± 0.48^#^ *(0.16–0.64)*	0.29 ± 0.61^#^ *(-0.02–0.59)*	0.31 ± 0.54 *(0.04–0.57)*	0.078^#^	0.170^#^	-0.12 ± 0.47^#^ *(-0.35–0.11)*	-0.09 ± 0.49^#^ *(-0.33–0.15)*	0.705^#^	0.47	0.48
Tip (°)											
DCT = +	4.31 ± 3.09^#^	3.72 ± 2.51	1.42 ± 3.12	0.69 ± 2.82	0.000^#,*^	0.000^*^	-2.89 ± 2.82^#^	-3.03 ± 1.87	0.647^#^	3.98	3.53
MCT = -	*(2.78–5.85)*	*(2.47–4.97)*	*(-0.13–2.97)*	*(-0.70–2.10)*			*(-4.29- -1.49)*	*(-3.95- -2.10)*			
Torque (°)											
BCT = +	*-0.14 ± 1.71*	*-1.00 ± 2.32*	*0.30 ± 1.97* ^*#*^	*1.34 ± 2.57*	0.286^#^	0.014^*^	*0.44 ± 1.80*	*2.33 ± 3.60* ^*#*^	0.048^#,*^	1.80	4.21
LCT = -	*(-0.99–0.71)*	*(-2.15–0.16)*	*(-0.68–1.28)*	*(0.06–2.61)*			*(-0.46–1.33)*	*(0.54–4.12)*			
Rotation (°)											
D-in = +	*1.74 ± 2.76*	*0.83 ± 1.59* ^*#*^	*1.76 ± 1.76*	*0.22 ± 1.86*	0.971	0.157^#^	*0.02 ± 2.10* ^*#*^	*-0.61 ± 1.89*	0.557^#^	2.04	1.94
M-in = -	*(0.37–3.11)*	*(0.04–1.62)*	*(0.89–2.64)*	*(-0.70–1.14)*			*(-1.02–1.06)*	*(-1.56–0.33)*			

*Abbreviation: VPa* power arm side of virtual model, *VC* control side of virtual model, *APa* power arm side of actual model, *AC* control side of actual model, *APa-VPa* Difference between actual and virtual model on power arm, *VC-AC* Difference between actual and virtual model on control

Favorable movement = + (Distalization, Extrusion, Buccal, Distal Crown Tip (DCT), Buccal Crown Torque (BCT), Distal-in rotation)

Non-Favorable movement =—(Mesialization, Intrusion, Lingual, Mesial Crown Tip (MCT), Lingual Crown Torque (LCT), Mesial-in rotation)

^#^non- parametric

^*^Statistically significant at *p* < 0.05

The difference in values between virtual and actual molar mesialization showed no significant differences between the PA (APa-VPa -0.58 ± 0.59 mm) and the control group (AC-VC -0.52 ± 0.59 mm). The RMSE indicated that the PA (0.81 mm) showed similar accuracy with the control group (0.77 mm).

### Maxillary molar tipping (Table 8)

The difference in values between the virtual and actual showed no significant differences between the PA (APa-VPa -2.89 ± 2.82°) and control (AC-VC -3.03 ± 1.87°). The RMSE indicated that the PA (3.98°) showed similar accuracy with the control group (3.53°).

### Error measurements

Results using Dahlberg’s formula revealed that all displacement and angular measurements did not exceed 0.5 mm/ ° for any of the investigated variables (Table 9).

**Table 9:** Error analysis using Dahlberg’s formula. (*N* = 6)

*Maxillary* *Tooth*	*Dahlberg’s value*					
	*Displacement*			*Angulation*		
	*A-P (mm)*	*Vertical (mm)*	*Transverse (mm)*	*Tip (°)*	*Torque (°)*	*Rotation (°)*
Canine	0.10	0.15	0.09	0.37	0.37	0.11
Premolar	0.11	0.11	0.06	0.31	0.21	0.30
First Molar	0.12	0.10	0.16	0.40	0.21	0.40

*Abbreviation: A-P* Anteroposterior

### Harm

Clear aligners are usually safe when used under the supervision of an orthodontist. However, our study identified potential risks and issues, such as off tracking, teeth not aligning properly due to tipping and rotation, which can lead to incomplete correction. The severity of these issues was mainly associated with non-compliance, including forgetting to wear the aligners, not wearing them as instructed, or failing to use elastics as directed. This can result in possible backtracking of the treatment process, the need for additional aligners, and extended treatment time. Nonetheless, these potential risks can be considered minor and reversible side effects.

## Discussion

**Fig. 6:**
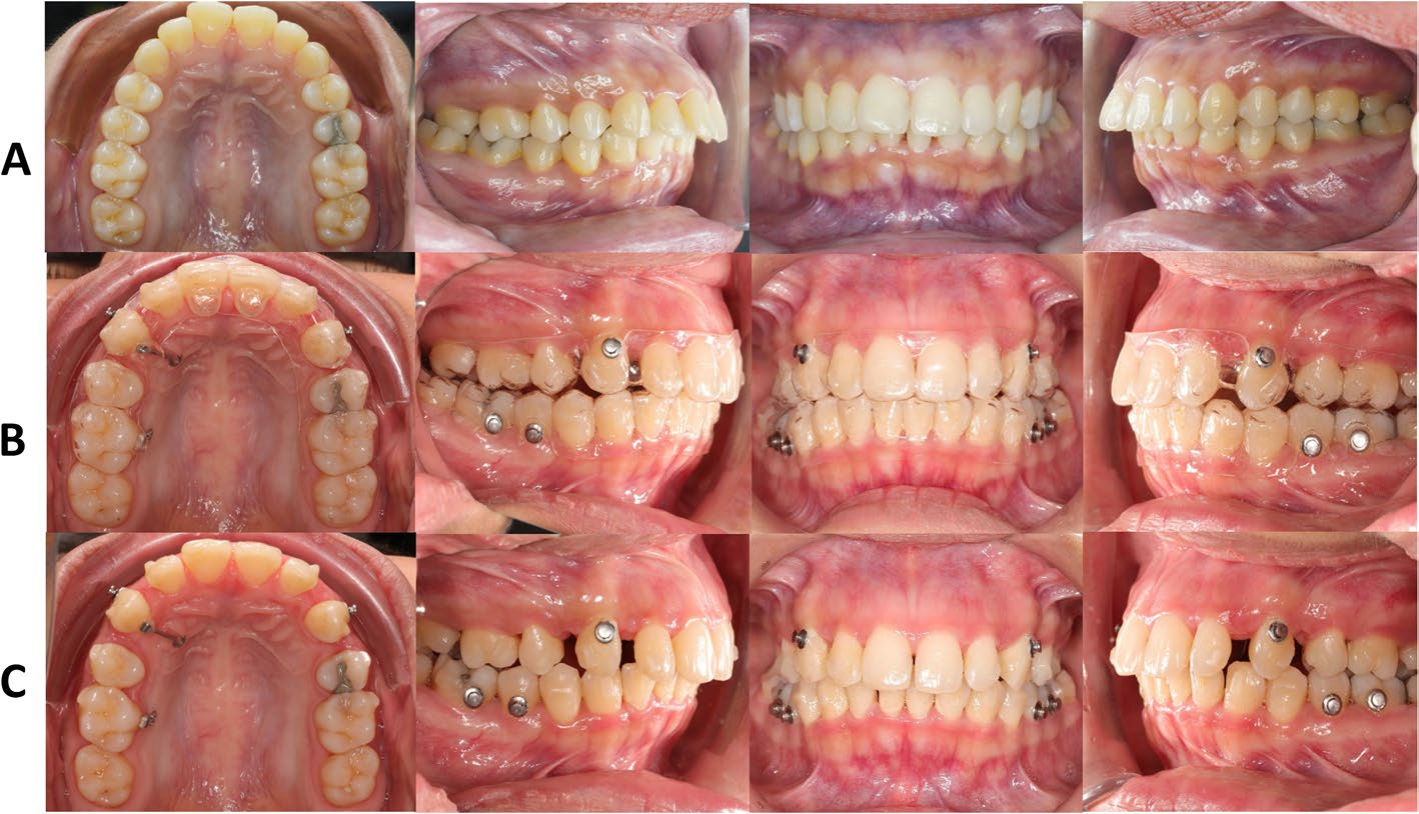
Intraoral photograph row A, pretreatment stage; B, in-house clear aligner at 24th aligners; C, stage 24th without aligners. Right side (quadrant 1) is power arm. Left side (quadrant 2) is control

To date, only seven published clinical papers using Invisalign® have evaluated the effectiveness of clear aligners in terms of tooth movement accuracy in premolar extraction cases [9, 14–17, 33, 34]. Our clinical research team was perhaps the first group to conduct a predictability study in premolar extraction cases using IHCA (Fig. 6).

What we found for maxillary canine was that both groups achieved distalization at approximately 60% of the extraction site. The difference amount in distalization between actual and virtual groups was also similar (APa-VPa 0.19 VS AC-VC 0.33 mm). In terms of predictability (RMSE), both groups showed similar deviation (PA 0.62/ C 0.67 mm). Likewise, we found that both the power arm and control groups could not achieve distal root uprighting as in the virtual plan. Both groups showed significant distal crown tipping (APa -11.10, AC -8.80°). An obviously similar increase in distal crown tipping was achieved in both the power arm and control groups (APa-VPa -10.43 / AC-VC -10.6°). In terms of predictability (RMSE), both groups showed similar deviation. the power arm group (PA 11.66/ C 11.50°). Furthermore, our study found that the actual group had significantly greater distal-in rotation than the virtual set-up (APa-VPa 3.54 VS AC-VC 11.57°). PA side showed significantly better accuracy in distal-in rotation (PA 7.85/ C 15.98°). However, PA did not increase the accuracy of maxillary canines in other tooth movement types.

In comparison with other studies, we found that the side effect of distal crown tipping in maxillary canines aligned with previous finite element studies in extraction cases [3, 4] and previous clinical studies using Invisalign [9, 14–17, 27]. Moreover, our findings may indicate that, for maxillary canine distalization and tipping, the palatal power arm did not enhance tooth movement accuracy nor reduce the difference between the predicted and achieved results. However, it appears to assist in decreasing distal-in rotation in maxillary canines. Unexpectedly, our study found that the actual canine was distalized slightly more than the virtual setup, particularly on the control side. The reasons for this could be (1) Class II elastics or (2) undercut block-out at the distal sides of the maxillary canine.

The observation that we still found the maxillary canine tipping indicates the need for more stringent protocols to control the root tipping of canines. For example, we could have increased the compensatory setup [16], enhanced plastic wrapping [20], used vertical attachment [15], and modified the design to increase stiffness in the edentulous area [35]. One finite element study demonstrated that various aligner designs for the extraction space- such as edentulous space, premolar pontic, half-sized premolar pontic, and rectangular column beam- significantly impacted the efficiency of space closure and force distribution on the canines. They found that the rectangular column beam design was the most effective in enhancing the local stiffness of the clear aligner in the edentulous area [35]. In contrast, our study employed a premolar pontic design with a reduced mesiodistal width, which we implemented at the beginning of treatment, followed by an edentulous design. Consequently, our aligner design may have lacked sufficient stiffness to effectively manage the distal tipping of the maxillary canine crown.

Regarding anchorage, we found that the actual group experienced significantly greater mesialization, intrusion, and mesial tipping compared to the virtual group. The mean virtual movement of premolar was programed to approach the pretreatment position (VPa 0.01 ± 0.67/ VC -0.08 ± 0.52 mm), the achieved mesialization of premolars in both power arm and control groups was similarly around 0.6 mm (APa -0.60 ± 0.49 mm / AC -0.59 ± 0.59 mm). Similarly, the mean virtual movement of molar was programmed to approach the pretreatment position (VPa 0.13 ± 0.58 / VC -0.08 ± 0.37 mm). However, the achieved medialization of molars in both power arm and control groups was around 0.5 mm (APa -0.45 ± 0.48 mm / AC -0.60 ± 0.53 mm). Likewise, the difference value between actual and virtual premolar depicted significant mesial tipping on both sides (APa -5.24 ± 3.47 / AC -3.829 ± 2.77°). and the molar experienced significant mesial tipping on both sides (APa -2.89 ± 2.82 / AC -3.03 ± 1.87°).

Our anchorage loss was similar to those in previous clinical studies [10, 14–17] and several FEM studies [10, 11]. Although various anchorage control strategies were applied in both the PA and control groups in our study, such as adding anti-mesial crown tipping [17], using attachments on second premolars and first molars [36], and implementing short class II elastics [37].

Our results indicated that, at the 24th IHCA, the PA did not effectively reduce distal crown tipping of the maxillary canines. Several factors might contribute to this phenomenon, such as inadequate height of the PA, insufficient plastic wrapping, and insufficient clear aligner wearing time, etc. [20]. The height of the palatal power arm was likely insufficient to counteract the significant tipping moment from clear aligner deflection at the extraction site. Additionally, employing short class II elastics might create a resultant force vector that passes below the center of resistance. Another concern was the inadequate wrapping of plastic around the canine at the proximal and the power arm bonding block-out area. Furthermore, a CA wearing time of one week may be too brief for the root of the canine to keep pace with the frequent activation of CA. We also hypothesized that by the 24th aligner, as the canine had been positioned in the middle of the extraction space, there may be less deflection of the clear aligner toward the extraction site. Therefore, the distal crown tipping of the power arm and control was equivalent. However, despite this, the palatal force from the power chain may effectively contribute to a more significant reduction in distal-in rotation of the canines in the power arm group.

Our study combined several strategies to counter the bowing effect and achieve maximum clinical anchorage. These strategies included a compensatory setup, overcorrections, movement staging by moving the canine first, followed by partial unraveling of the incisors (frog pattern), adding an exaggerated curve of Spee, attachments, short class II intermaxillary elastics, and PA on the experimental side [15, 20–22] Despite these efforts, bodily distalization of the canine and maximum anchorage were not fully attained in our study, which contrasted with the findings of Johal and Bondemark [38] and Inan and Gonca [39], who proposed using power arms to apply force closer to the center of resistance, theoretically minimizing the distal tipping of the canine.

### Strength

The main strength of our study was the RCT with a split-mouth design. Additionally, the subjects’ characteristics at pretreatment, intervention, comparison, and outcome (PICO) principles were specifically employed. For the inspection of 3D deviation, we used GOM Inspect software, known for its high precision in the industry. The software automatically linked the anatomy of each crown element at pretreatment and the 24th IHCA, thereby eliminating human error.

### Limitations

This study has some notable limitations. The off-track problems in clear aligner treatment that arise when the teeth do not move as intended can impact the measured outcomes. Additionally, individual responses to IHCA treatment may vary due to differences in dental anatomy and supporting structures. Furthermore, the type of material impacts the clinical performance of clear aligners [40]. Therefore, it is essential to avoid applying characteristics identified for one aligner material to others in a generalized way, especially when comparing Invisalign (Thermoplastic Polyurethane, TPU) with PETG (Polyethylene Terephthalate Glycol, PETG) [2, 40, 41]. The primary reason for using PETG in our study was the lack of a TPU distributor in our country at the time we conducted the research.

Moreover, specific aligner designs and thicknesses affect performance. For example, Lyu et al. [42] conducted a finite element analysis with 0.75 mm thick aligners, finding that a 2 mm extension of plastic below the gingival margin resulted in greater posterior tooth displacement and improved tipping control compared to others designs.

### Generalizability

The generalizability of this study is limited due to variations in laboratory and clinical protocols. Additionally, our study results were obtained during the mid-phase of canine retraction, whereas other Invisalign studies reported outcomes at the completion of space closure.

### Clinical recommendations

IHCA could be utilized for the canine retraction phase of maxillary premolar extraction cases. However, it may present unique challenges, as the treatments require a more complex aligner design to control tooth movements with maximum anchorage. Including a palatal power arm may not significantly improve aligner accuracy during the middle phase of maxillary canine retraction. This indicates that employing concurrent strategies, which include multi-stage scanning, compensatory overcorrection, sequential space closure, proper attachments, and frequent monitoring, may be equally effective. Future research suggestions may involve prospective studies with larger sample sizes. Additionally, superimposition using 3D cone-beam computed tomography should be incorporated.

## Conclusions

1. IHCA could be utilized for the canine retraction phase of maxillary premolar extraction cases.
2. Maxillary canines exhibited significantly greater distal crown tipping and distal-in rotation compared to the virtual setup.
3. According to the RMSE, the palatal power arm may not control the maxillary canine more effectively than the control group, but it may provide better rotational control.
4. Anchorage: maxillary second premolars and first molars display mesialization, mesial crown tipping, intrusion that was greater than predicted.
5. Both the power arm and control groups experienced a similar loss of anchorage.

## Data Availability

Data will be available upon request.
